# Development of an LLM pipeline exceeding physician-documented cardiovascular risk scores under routine clinical conditions

**DOI:** 10.1093/ehjdh/ztag124

**Published:** 2026-07-28

**Authors:** Tobias Roeschl, Marie Hoffmann, Axel Unbehaun, Henryk Dreger, Gerhard Hindricks, Volkmar Falk, Ran Balicer, Radu Tanacli, Felix Hohendanner, Alexander Meyer

**Affiliations:** Deutsches Herzzentrum der Charité, Department of Cardiology, Angiology and Intensive Care Medicine, Charitéplatz 1, Berlin 10117, Germany; Charité – Universitätsmedizin Berlin, corporate Member of Freie Universität Berlin and Humboldt-Universität zu Berlin, Charitéplatz 1, Berlin 10117, Germany; Charité – Universitätsmedizin Berlin, Institute for Artificial Intelligence in Medicine, Invalidenstraße 120, Berlin 10115, Germany; Berlin Institute of Health at Charité – Universitätsmedizin Berlin, Charitéplatz 1, Berlin 10117, Germany; DZHK (German Centre for Cardiovascular Research), partner Site Berlin, Potsdamer Str. 58, 10785 Berlin, Germany; Deutsches Herzzentrum der Charité, Department of Cardiothoracic and Vascular Surgery, Augustenburger Platz 1, Berlin 13353, Germany; Charité – Universitätsmedizin Berlin, corporate Member of Freie Universität Berlin and Humboldt-Universität zu Berlin, Charitéplatz 1, Berlin 10117, Germany; Charité – Universitätsmedizin Berlin, Institute for Artificial Intelligence in Medicine, Invalidenstraße 120, Berlin 10115, Germany; DZHK (German Centre for Cardiovascular Research), partner Site Berlin, Potsdamer Str. 58, 10785 Berlin, Germany; Deutsches Herzzentrum der Charité, Department of Cardiothoracic and Vascular Surgery, Augustenburger Platz 1, Berlin 13353, Germany; Charité – Universitätsmedizin Berlin, corporate Member of Freie Universität Berlin and Humboldt-Universität zu Berlin, Charitéplatz 1, Berlin 10117, Germany; Deutsches Herzzentrum der Charité, Department of Cardiothoracic and Vascular Surgery, Augustenburger Platz 1, Berlin 13353, Germany; Charité – Universitätsmedizin Berlin, corporate Member of Freie Universität Berlin and Humboldt-Universität zu Berlin, Charitéplatz 1, Berlin 10117, Germany; Deutsches Herzzentrum der Charité, Department of Cardiology, Angiology and Intensive Care Medicine, Augustenburger Platz 1, Berlin 13353, Germany; Deutsches Herzzentrum der Charité, Department of Cardiology, Angiology and Intensive Care Medicine, Charitéplatz 1, Berlin 10117, Germany; Charité – Universitätsmedizin Berlin, corporate Member of Freie Universität Berlin and Humboldt-Universität zu Berlin, Charitéplatz 1, Berlin 10117, Germany; Charité – Universitätsmedizin Berlin, corporate Member of Freie Universität Berlin and Humboldt-Universität zu Berlin, Charitéplatz 1, Berlin 10117, Germany; DZHK (German Centre for Cardiovascular Research), partner Site Berlin, Potsdamer Str. 58, 10785 Berlin, Germany; Deutsches Herzzentrum der Charité, Department of Cardiothoracic and Vascular Surgery, Augustenburger Platz 1, Berlin 13353, Germany; Translational Cardiovascular Technologies, Institute of Translational Medicine, Department of Health Sciences and Technology, Swiss Federal Institute of Technology, Zürich 8092, Switzerland; Charité – Universitätsmedizin Berlin, corporate Member of Freie Universität Berlin and Humboldt-Universität zu Berlin, Charitéplatz 1, Berlin 10117, Germany; Innovation Division, Clalit Health Services, Clalit Research Institute, Tel Aviv, Israel; Charité – Universitätsmedizin Berlin, corporate Member of Freie Universität Berlin and Humboldt-Universität zu Berlin, Charitéplatz 1, Berlin 10117, Germany; Deutsches Herzzentrum der Charité, Department of Cardiology, Angiology and Intensive Care Medicine, Augustenburger Platz 1, Berlin 13353, Germany; Deutsches Herzzentrum der Charité, Department of Cardiology, Angiology and Intensive Care Medicine, Charitéplatz 1, Berlin 10117, Germany; Charité – Universitätsmedizin Berlin, corporate Member of Freie Universität Berlin and Humboldt-Universität zu Berlin, Charitéplatz 1, Berlin 10117, Germany; Deutsches Herzzentrum der Charité, Department of Cardiology, Angiology and Intensive Care Medicine, Augustenburger Platz 1, Berlin 13353, Germany; Charité – Universitätsmedizin Berlin, corporate Member of Freie Universität Berlin and Humboldt-Universität zu Berlin, Charitéplatz 1, Berlin 10117, Germany; Charité – Universitätsmedizin Berlin, Institute for Artificial Intelligence in Medicine, Invalidenstraße 120, Berlin 10115, Germany; Berlin Institute of Health at Charité – Universitätsmedizin Berlin, Charitéplatz 1, Berlin 10117, Germany; DZHK (German Centre for Cardiovascular Research), partner Site Berlin, Potsdamer Str. 58, 10785 Berlin, Germany; Berlin Institute for the Foundations of Learning and Data – TU Berlin, Berlin, Germany

**Keywords:** Large language models, Clinical workflow automation, Knowledge retrieval, Clinical information extraction, Cardiovascular risk scores, Digital health

## Abstract

**Aims:**

Risk scores are essential to evidence-based cardiovascular care, but manual calculation is labour intensive and error prone. Large language models (LLMs) could automate this process, yet LLMs are limited by their propensity for calculation errors and factual hallucinations. Pipelines separating LLM-based data extraction from deterministic score computation may improve reliability and transparency.

**Methods and results:**

We conducted a retrospective diagnostic study at a quaternary heart centre in Germany (January 2020 to July 2023). Patients with atrial fibrillation (*n* = 179) from an ablation registry and patients with severe aortic stenosis (*n* = 76) evaluated by a heart team were included. Six LLMs (GPT-5.2, Gemini 3.1 Pro, DeepSeek-R1, Qwen3, GPT-OSS 120B, and Kimi K2.5) were tested in standalone, retrieval-augmented generation (RAG), and pipeline configurations to compute HAS-BLED, CHA_2_DS_2_-VASc, and EuroSCORE II scores from routine clinical reports. Accuracy was assessed against expert-adjudicated ground truth using root mean squared error (RMSE) and Krippendorff’s α to evaluate numerical deviation and categorical agreement, respectively. Pipeline-generated scores showed substantially higher agreement with expert adjudication than standalone LLMs, LLMs with RAG, and treating physicians (mean Krippendorff’s α: 0.78 vs. 0.32 vs. 0.39 vs. 0.31) and lower deviation from ground truth (mean RMSE: 0.89 vs. 5.81 vs. 1.85 vs. 1.34).

**Conclusion:**

Pipelines combining expert-curated knowledge injection, LLM-based clinical data extraction, and deterministic score calculation enable accurate and scalable cardiovascular risk score computation from unstructured real-world clinical data, outperforming physician-documented scores. Such pipelines could form the basis for clinical decision-support systems that automate routine risk assessment, reduce clinician workload, and promote more consistent evidence-based care.

## Graphical Abstract

**Graphical Abstract:**
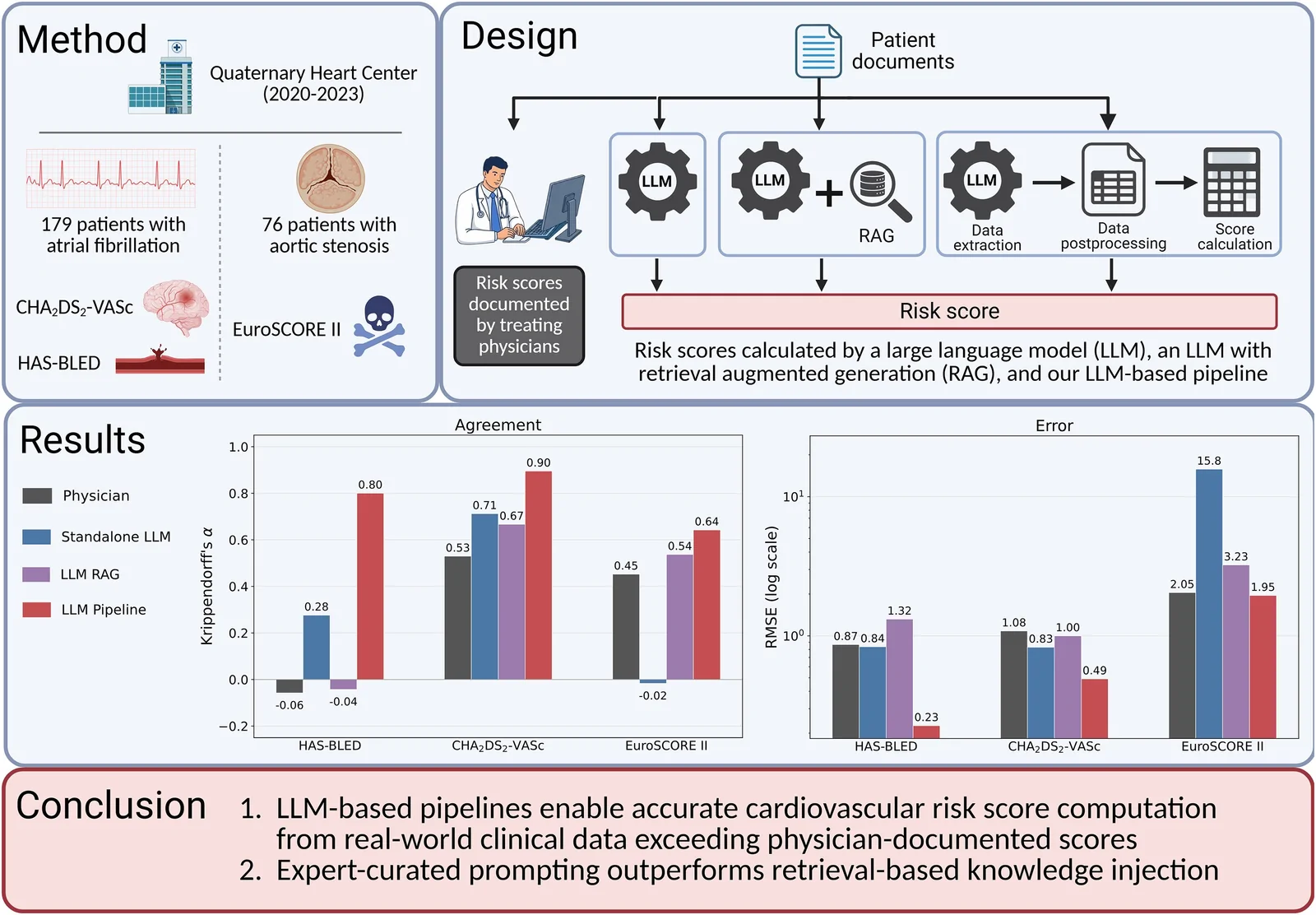

## Introduction

Clinical risk scores are foundational instruments in medicine, translating complex patient data into actionable risk estimates that guide therapeutic decisions and support guideline-adherent care. As medicine becomes increasingly data driven,^1^ accurate calculation of these scores is vital. Typically, physicians must manually extract variables from unstructured electronic health records—a workflow that is both laborious and susceptible to error.

Large language models (LLMs) could automate such text-heavy clinical tasks, yet their application to quantitative risk scoring is fundamentally undermined by well-documented limitations. These models are prone to factual and contextual hallucinations^2^—generating confident but incorrect outputs—and often exhibit poor arithmetic reasoning,^3–5^ limitations that are particularly hazardous where patient safety and guideline adherence are at stake. While recent work^6^ has shown that integrating LLMs with external calculators can improve numerical accuracy, a critical translational gap persists. Previous studies have relied on synthetic vignettes or concise case reports, which do not reflect the complexity and noise of authentic clinical documents such as multi-page discharge letters or imaging reports.^3,6–8^ Performance of these systems in a real-world clinical setting therefore remains unknown.

To date, no study has validated an LLM-based system for cardiovascular risk score calculation on authentic, unstructured patient reports and rigorously benchmarked its performance against treating physicians.

In our study, we aimed to address these gaps by evaluating how accurately standalone LLMs, LLMs with retrieval-augmented generation (RAG^9^) and LLM-based pipelines compute cardiovascular risk scores from routine clinical text reports compared to treating physicians. The LLM-based pipeline we developed integrates the language-understanding capabilities of LLMs with the reliability of deterministic computation. It comprises three stages: (i) expert-curated, guideline-concordant score definitions, (ii) LLM-driven extraction of relevant data from original clinical reports, and (iii) a separate, formula-based engine for the final score computation.

We conducted this comparison using three widely adopted cardiovascular risk scores—CHA_2_DS_2_-VASc,^10^ HAS-BLED,^11^ and EuroSCORE II^12^—across two patient cohorts. Our findings show that pipeline models achieve superior accuracy and calibration, outperforming standalone LLMs, the RAG approach, and treating physicians. These findings demonstrate the potential of this approach as a robust and operationally scalable framework for automated clinical risk assessment.

## Methods

### Study design and patient cohorts

Our retrospective study included two distinct patient cohorts from a single quaternary care heart centre. The first cohort included 179 of 182 consecutively screened adult patients from an in-house registry who underwent catheter ablation for atrial fibrillation (AF) between January 2020 and July 2023, each with a digitized discharge letter. The second cohort included 76 of 90 adults with severe aortic stenosis (AS) evaluated by one of our institutional heart teams during 2022, each with comprehensive digitized documentation. For the AS cohort, sufficient documentation was defined as the availability of at least one physician letter, echocardiography report, cardiac catheterization report, computed tomography (CT) report, and a heart team protocol. Patient characteristics for both cohorts are detailed in Supplementary material online, *Table S1* and Supplementary material online, *Table S2*. The study protocol was approved by the research ethics committee of Charité – Universitätsmedizin Berlin (EA2/183/23).

### Data acquisition and preparation

All clinical documents were extracted as PDF files from the hospital information system and converted into plain text using optical character recognition (OCR). The OCR output was manually reviewed to ensure fidelity to the original documents, particularly for clinically relevant information. In rare cases where OCR-related errors affecting relevant variables were identified, the text was manually corrected prior to model inference. For each patient in the AF cohort, the physician letter following the ablation procedure was used as the input document. For the AS cohort, a comprehensive single document was created for each patient by concatenating their two most recent discharge letters, the invasive coronary angiography report, echocardiography report, and CT scan report. This approach was used to simulate a complete chart review.

All reports were manually anonymized by replacing identifiable information with synthetic data, while preserving the original structure and clinical complexity. To maintain temporal relationships between events, all dates for a given patient were shifted by a randomly sampled offset.

We manually extracted risk scores documented by treating clinicians in discharge letters and heart team protocols to establish a reference standard for benchmarking. These values were then removed from the documents provided to the LLMs to prevent data leakage.

### Model architectures and prompting strategies

**Figure 1:**
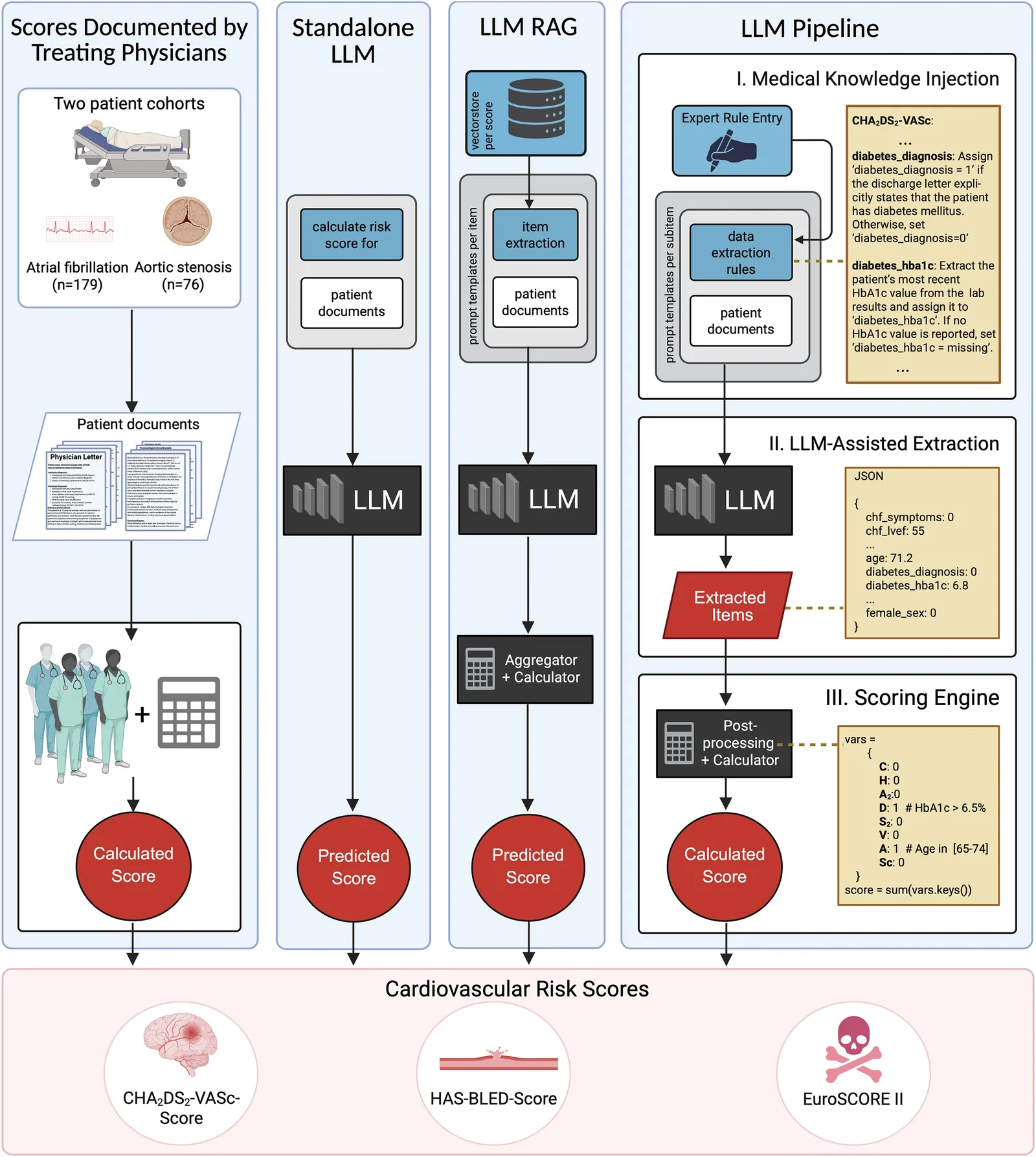
Experimental design. Four experimental arms evaluate risk score derivation in two patient cohorts based on original patient documents. Left: Risk scores documented by treating physicians during clinical practice. Middle left: In the standalone large language model (LLM) approach, a single prompt is used to directly predict the risk score end-to-end from patient documents. Middle right: retrieval augmented generation was used to provide contextual information for score-relevant data extraction prior to aggregation and calculation. Right: in the LLM pipeline, a three-stage process is applied: Stage I, expert-defined medical knowledge and extraction instructions were incorporated into prompts to enable granular data extraction; Stage II, the LLM outputs structured values for each variable; and Stage III, a deterministic scoring engine derives aggregated component values and applies validated formulas to compute the final risk score.

*Figure 1* provides a comprehensive overview of the three approaches evaluated: the standalone LLM approach, the LLM RAG approach, and the LLM pipeline approach.

In the standalone LLM approach, we instructed LLMs to calculate scores directly from clinical reports with reference only to the original score publications. In a single, comprehensive prompt, the full clinical document was provided alongside the instruction to calculate the complete risk score according to the respective original publication and output both the final score and all contributing score components in a structured JSON format. In the pipeline approach, risk score computation was organized into three stages: (i) provision of score component definitions, including surrogate parameters, enhanced by domain-specific knowledge incorporated into all prompt templates to integrate clinical expertise (e.g. symptom interpretation criteria) and specifications from the original score publications; (ii) LLM-based extraction of structured data from clinical reports; and (iii) deterministic calculation of the final score. The inclusion of surrogate parameters beyond the classical score components allowed for the integration of implicit knowledge into the score calculation by approximating how clinicians extract and interpret data in routine practice, thereby improving the accuracy of score component ascertainment and overall risk estimation. For example, the models were prompted to use the most recent HbA1c value to infer the presence of diabetes mellitus and assign a corresponding point in the CHA_2_DS_2_-VASc score, even when the diagnosis was not explicitly mentioned in the discharge letter. To improve extraction accuracy, we issued a separate query for each individual score component. A core prompt template was used for each score component across all models. Prompt templates are provided in the Supplementary material. As in the standalone LLM approach, we instructed the LLMs to produce structured JSON output. We developed a deterministic scoring engine, coded in Python, that used a rule-based approach to further process the extracted data and apply logical and arithmetic operations to compute the final score.

In the LLM RAG approach, we aimed to investigate whether data extraction could be guided by dynamically retrieved evidence from original score publications and related literature, rather than expert-derived, hand-crafted instructions as in the pipeline-based approach, thereby providing a more scalable alternative. Thus, we implemented the RAG approach at the component level, issuing a separate minimal query for each score component and augmenting it with dynamically retrieved evidence, without extracting additional surrogate parameters. The extracted component-level outputs were subsequently aggregated, and the final scores were deterministically calculated, consistent with the final processing steps of the pipeline-based approach. The score-specific literature corpora were curated using five criteria: (i) original score publications, (ii) validation and follow-up studies, (iii) major guideline documents, (iv) official calculator and model-definition sources, and (v) high-quality review articles. Separate FAISS vector stores were built for each score (chunk size 1000, chunk overlap 150), and evidence was retrieved by k-nearest-neighbour similarity search. When model/provider support was available, retrieval was exposed as a callable function tool (search_corpus) in tool-RAG mode; otherwise, retrieval was executed directly against FAISS and passed to the model as prompt context (prompt-RAG mode). In the current batch configuration, top-k retrieval was set to *k* = 5 (the orchestrator default is *k* = 8). The sources used for the RAG-based approach, categorized by score, are detailed in Supplementary material online, *Table S3*.

Across all three approaches, we tested six different LLMs: three optimized for reasoning (Qwen3-235B-A22B, DeepSeek-R1, and Kimi K2.5) and three leading models (GPT-5.2, Gemini 3.1 Pro, and GPT-OSS 120B). All prompts were executed in a zero-shot configuration with the temperature parameter set to zero to minimize output variation and enhance reproducibility.

All models received identical German input text and identical English prompt templates, ensuring a consistent and fair evaluation across models.

### Ground truth adjudication process

To establish a reference standard for all risk scores, two senior resident cardiologists independently reviewed all patient documents for both cohorts, blinded to the scores documented by the treating physicians. The cardiologists used a standardized data collection form detailing the definitions for every component of the HAS-BLED, CHA_2_DS_2_-VASc, and EuroSCORE II scores, as specified in their original publications (see Supplementary material online, *Table S4*). Raters were instructed to use the most recent value for any duplicate entries and to assume normal reference ranges for missing laboratory values. A standardized mapping was used for qualitative descriptions of left-ventricular ejection fraction (e.g. ‘moderately impaired’ was mapped to 35%). Any disagreements between the two primary raters were resolved by a third, independent senior cardiologist to reach a final consensus for each score component. Ground truth patient ages were programmatically calculated based on the date of birth and either the date of discharge or the date of the heart team meeting. Ground truth scores were calculated from these components using the score points and regression coefficients reported in the original publications, respectively.

To evaluate agreement between clinical raters during the establishment of ground truth values for score components, we employed Krippendorff’s α, using the nominal or ordinal variant as appropriate based on the measurement level of each variable. Due to the instability or inapplicability of these metrics for instances with sparse data, we additionally reported raw agreement as the proportion of identical ratings between raters. After consensus ground truth scores were established, we evaluated the performance of standalone LLMs, LLMs with RAG, pipeline models and physician-derived scores against these reference standards.

### Performance metrics and statistical analysis

Patient characteristics were reported as counts and percentages for binary variables, as means with 95% confidence intervals for normally distributed continuous variables, and as medians with interquartile ranges for non-normally distributed variables. Normality was assessed using the Shapiro–Wilk test.

We evaluated agreement between risk scores derived by the standalone LLMs, LLMs with RAG, the pipeline models, and treating physicians, and the ground truth scores across clinically relevant score categories. Specifically, we used the following threshold groupings: <2 vs. ≥2 for CHA_2_DS_2_-VASc,^13^ <3 vs. ≥3 for HAS-BLED,^11^ and <4%, 4–8%, >8% for EuroSCORE II.^14^ Agreement across these categories was assessed using Krippendorff's α, excluding instances with missing data. Prediction error was quantified using the root mean squared error (RMSE) between each derived score and the ground truth.

Calibration was evaluated using kernel density estimation (KDE) plots, with ground truth scores on the x-axis and predicted scores on the y-axis. The diagonal dashed line indicates perfect calibration, with density contours closer to this line reflecting better calibration.

Per-component accuracies, defined as the proportion of correctly assigned values for each score component, were visualized using radar plots. For the age variable, predictions within ±1 year were counted as correct for this metric.

All statistical analyses were conducted using Python version 3.9, scikit-learn 1.6, numpy 1.26, and krippendorff 0.8.

## Results

### Standalone LLMs do not ensure valid risk assessments for complex risk scores

**Figure 2:**
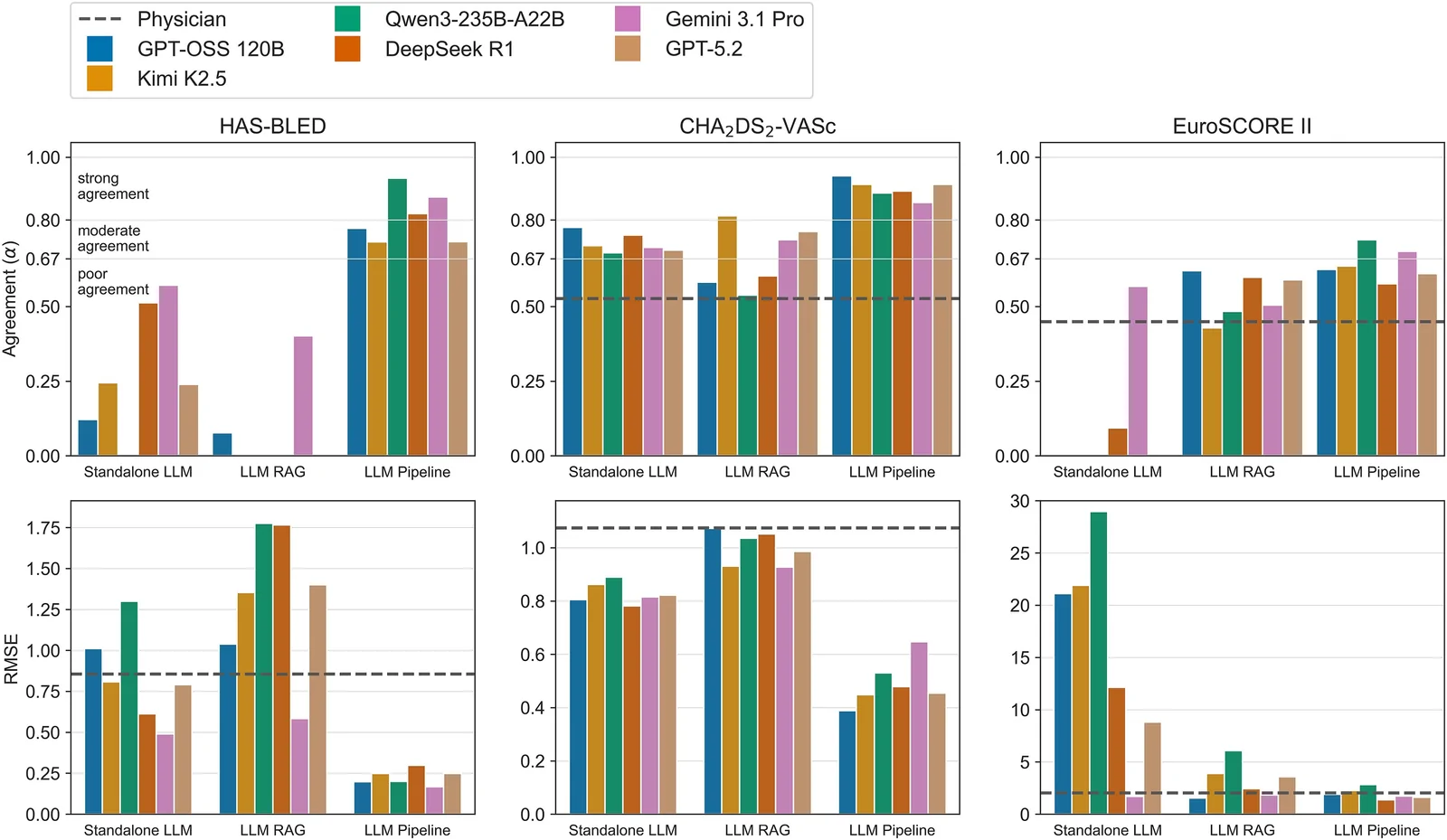
Agreement and error. Bar plots represent Krippendorff's αs and root mean squared errors for scores derived by treating physicians, standalone large language models (LLMs), LLMs with retrieval augmented generation, and LLM pipelines, respectively. Agreement was assessed based on clinically relevant risk score categories i.e. <2 vs. ≥2 for CHA_2_DS_2_-VASc,^13^ <3 vs. ≥3 for HAS-BLED,^11^ and <4%, 4–8%, >8% for EuroSCORE II.^14^ For the agreement plots, values below zero were truncated and therefore not displayed. Consequently, negative agreement estimates, including the Krippendorff’s α for the HAS-BLED score between treating physicians and the ground truth (α = −0.06), are not visible in the figure, but are reported in Supplementary material online, *Table S5*.

Applying these evaluation metrics, the standalone LLMs tasked with end-to-end score calculation produced inconsistent and often erroneous results. While many LLMs matched or exceeded the accuracy of treating physicians for the simpler additive HAS-BLED and CHA_2_DS_2_-VASc scores, most models failed to correctly calculate the more complex EuroSCORE II, which is derived from a logistic regression model applying the logistic function to a weighted linear combination of clinical variables. This failure was evident in the extremely high RMSE values and poor agreement for EuroSCORE II scores derived by most standalone LLMs (*Figure 2*, Supplementary material online, *Table S5*). Calibration plots suggest that most tested LLMs systematically overestimated EuroSCORE II values relative to ground truth (see Supplementary material online, *Figure S1*). These findings confirm that standalone LLMs are currently not reliable for complex clinical calculations from real-world data.

### Knowledge conflicts and algebraic limitations undermine LLM accuracy for complex risk scores

**Figure 3:**
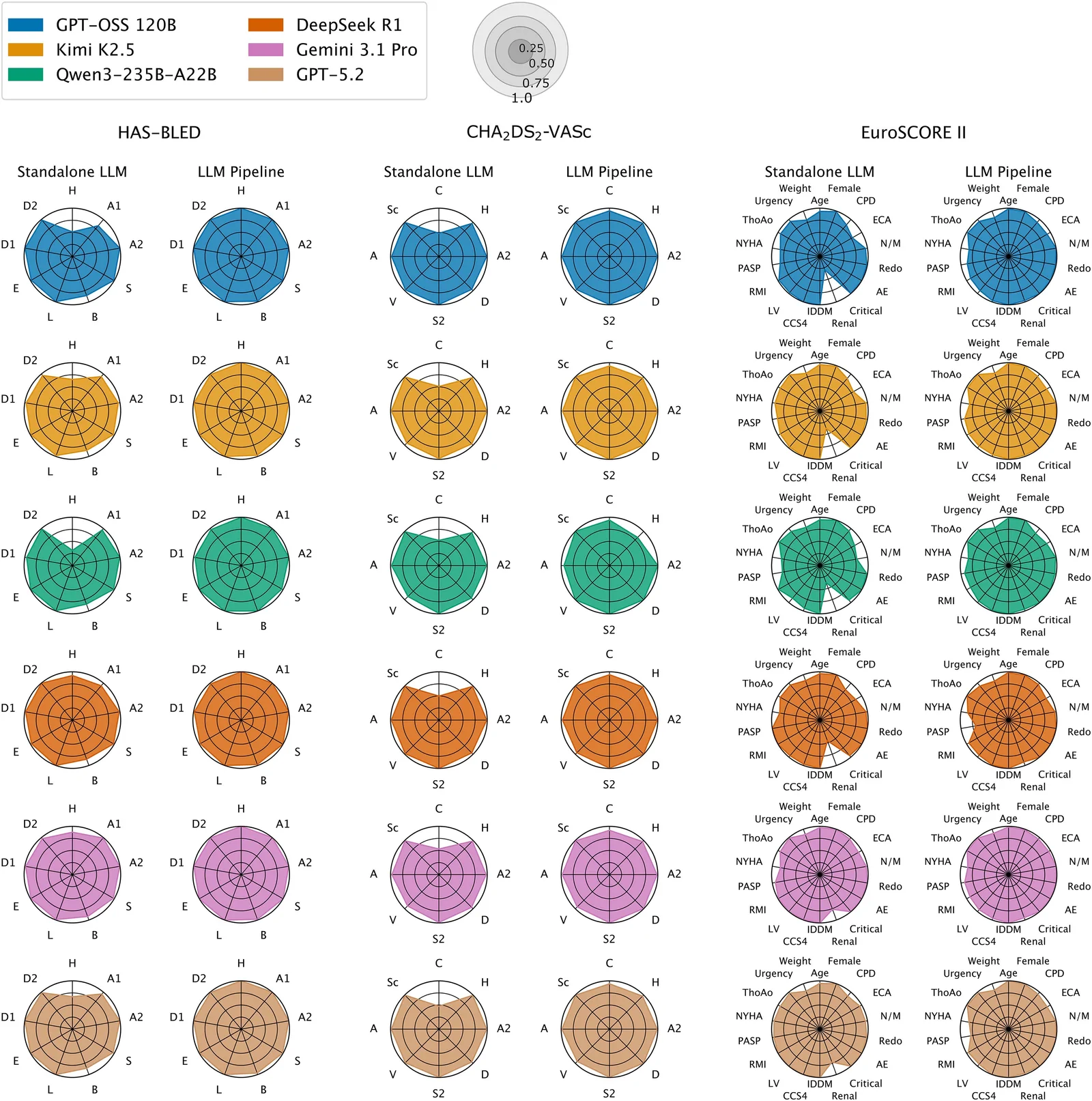
Per-component accuracy for standalone large language models (LLMs) compared to LLM pipelines. Radar plots represent mean accuracies for individual score components. Mean accuracies across all LLMs and components are 0.930 (HAS-BLED), 0.917 (CHA_2_DS_2_-VASc), and 0.919 (EuroSCORE II) for the standalone LLM, and 0.993 (HAS-BLED), 0.974 (CHA_2_DS_2_-VASc), and 0.950 (EuroSCORE II) for the LLM pipeline approach. Score component abbreviations are shown in Supplementary material online, *Table S4*.

Analysis of per-component accuracies of standalone LLMs (*Figure 3*) revealed two primary performance limitations.

First, LLMs often relied on their internal, generalized definitions of medical terms instead of the specific criteria used in the original score publications, a phenomenon known as knowledge conflict^15^ or contextual hallucination.^2^ For instance, models frequently assigned a point for ‘Hypertension’ in the HAS-BLED score even when the patient’s blood pressure was well controlled and did not meet the score’s specific criterion of systolic blood pressure >160 mmHg (see Supplementary material online, *Table S4*). As shown in the radar plots (*Figure 3*, Supplementary material online, *Figure S2*), this led to poor per-component accuracies for several variables.

Second, standalone LLMs demonstrated significant score-complexity-dependent algebraic limitations. This was suggested by the observation that high per-component accuracies (see Supplementary material online, *Figure S2*, Supplementary material online, *Figure S3*, Supplementary material online, *Figure S4*) did not always translate into accurate final-score predictions, as reflected in RMSE and categorical agreement. To assess the arithmetic capabilities of standalone LLMs in score calculation, we compared the final scores directly reported by the models with scores recalculated deterministically from the score components reported in the same outputs. For the two additive point-based scores, arithmetic consistency was generally high, particularly for CHA_2_DS_2_-VASc, where exact agreement between reported and recalculated scores exceeded 97% for all models (see Supplementary material online, *Figure S5*). In contrast, consistency was poor for EuroSCORE II for most models, with exact agreement ranging from 0.0% to 15.8% for five of six models. Gemini 3.1 Pro was a notable exception, achieving substantially higher arithmetic consistency for EuroSCORE II than the other standalone models. However, because a relevant proportion of its final EuroSCORE II predictions were still not reproducible from its own reported components, strong aggregate performance did not always correspond to traceable and auditable risk score generation. Taken together, these findings indicate that standalone LLMs can often aggregate simple additive scores, but struggle to perform more complex regression-based score calculations reliably.

### LLM-based pipelines overcome LLM shortcomings in algebraic reasoning

**Figure 4:**
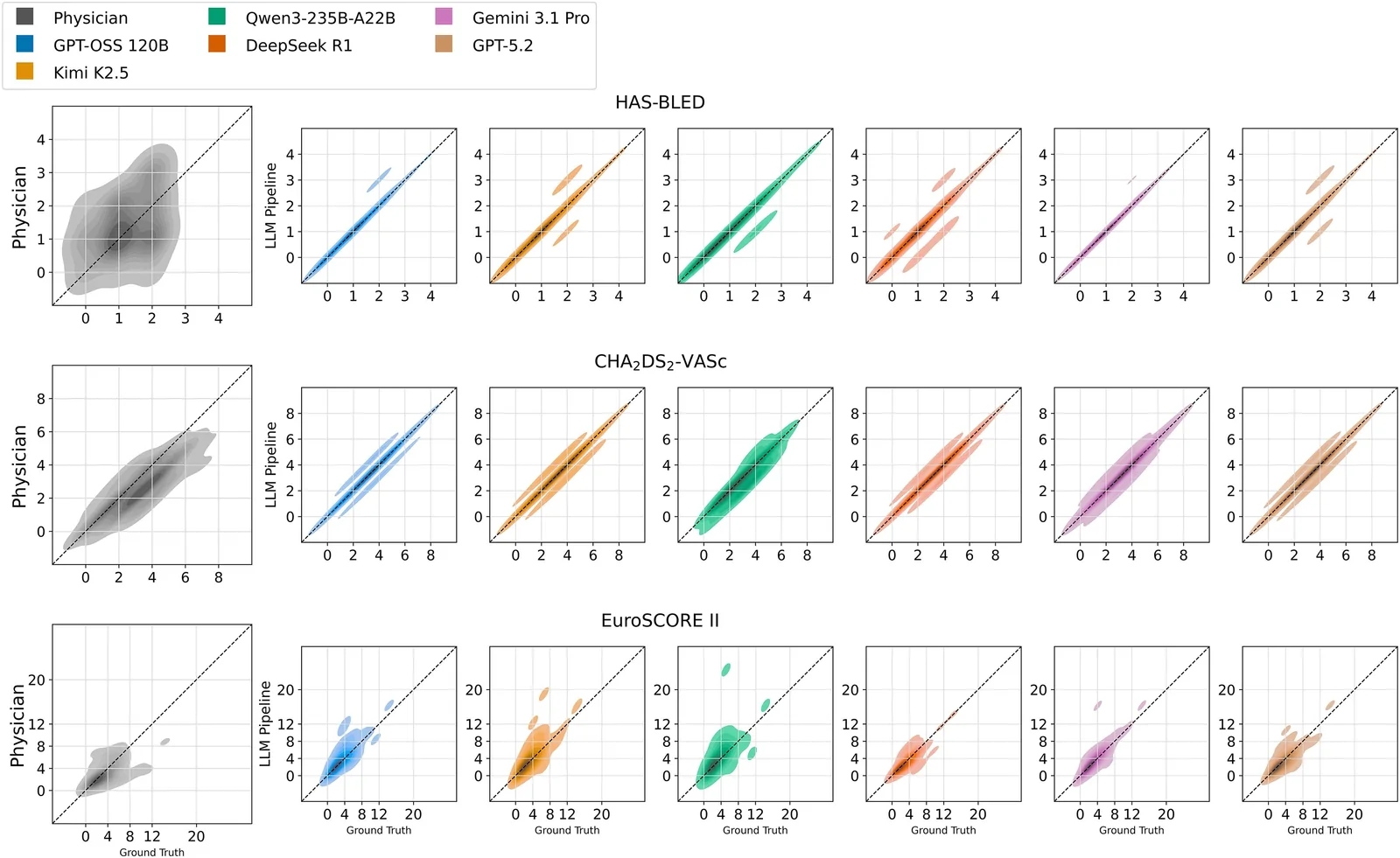
Calibration of physician-derived scores and pipeline model-derived scores. Calibration was visualized using kernel density estimation plots, with the ground truth score on the x-axis and the predicted score on the y-axis. The diagonal dashed line indicates perfect calibration. Densities closer to this line reflect better calibration between predicted and true scores.

Our pipeline model, which decouples data extraction from score calculation, successfully mitigated these performance limitations. Instructing the LLMs to extract only granular data points (e.g. laboratory values, dates, medication data) and delegating all arithmetic and logical operations to a deterministic downstream algorithm substantially improved performance. For most LLMs tested, the pipeline approach clearly outperformed standalone LLMs, the RAG approach and treating physicians across all risk scores and evaluation metrics. Agreement with the ground truth in clinically relevant categories was markedly higher for the pipeline models compared to scores derived by standalone LLMs, LLMs with RAG, and scores documented by treating physicians (mean Krippendorff’s α across all scores: 0.78 vs. 0.32 vs. 0.39 vs. 0.31). Similarly, the pipeline approach showed the lowest overall deviation from ground truth (mean RMSE across all LLMs and scores: 0.89 vs. 5.81 vs. 1.85 vs. 1.34) and demonstrated superior calibration compared to standalone LLMs (see Supplementary material online, *Figure S1*), LLMs with RAG (see Supplementary material online, *Figure S6*) and treating physicians (*Figure 4*). Notably, treating physicians tended to underestimate patient risk compared to the expert-adjudicated ground truth (*Figure 4*, Supplementary material online, *Table S6*).

### Current LLMs are reliable data extractors but may be poor medical data interpreters

Despite the pipeline’s high performance, per-component accuracies remained comparatively lower for EuroSCORE II components that require nuanced clinical interpretation, such as NYHA class. This suggests that while LLMs are reliable at structured data extraction, they are less adept at interpreting ambiguous clinical context. However, these same score components also exhibited the lowest interrater agreement among the expert cardiologists who established the ground truth (see Supplementary material online, *Table S7*). This finding highlights that the challenge in evaluating such variables is rooted in the interpretive nature of their clinical definitions, affecting both humans and artificial intelligence.

### LLM-based pipelines show promise in treatment optimization

**Figure 5:**
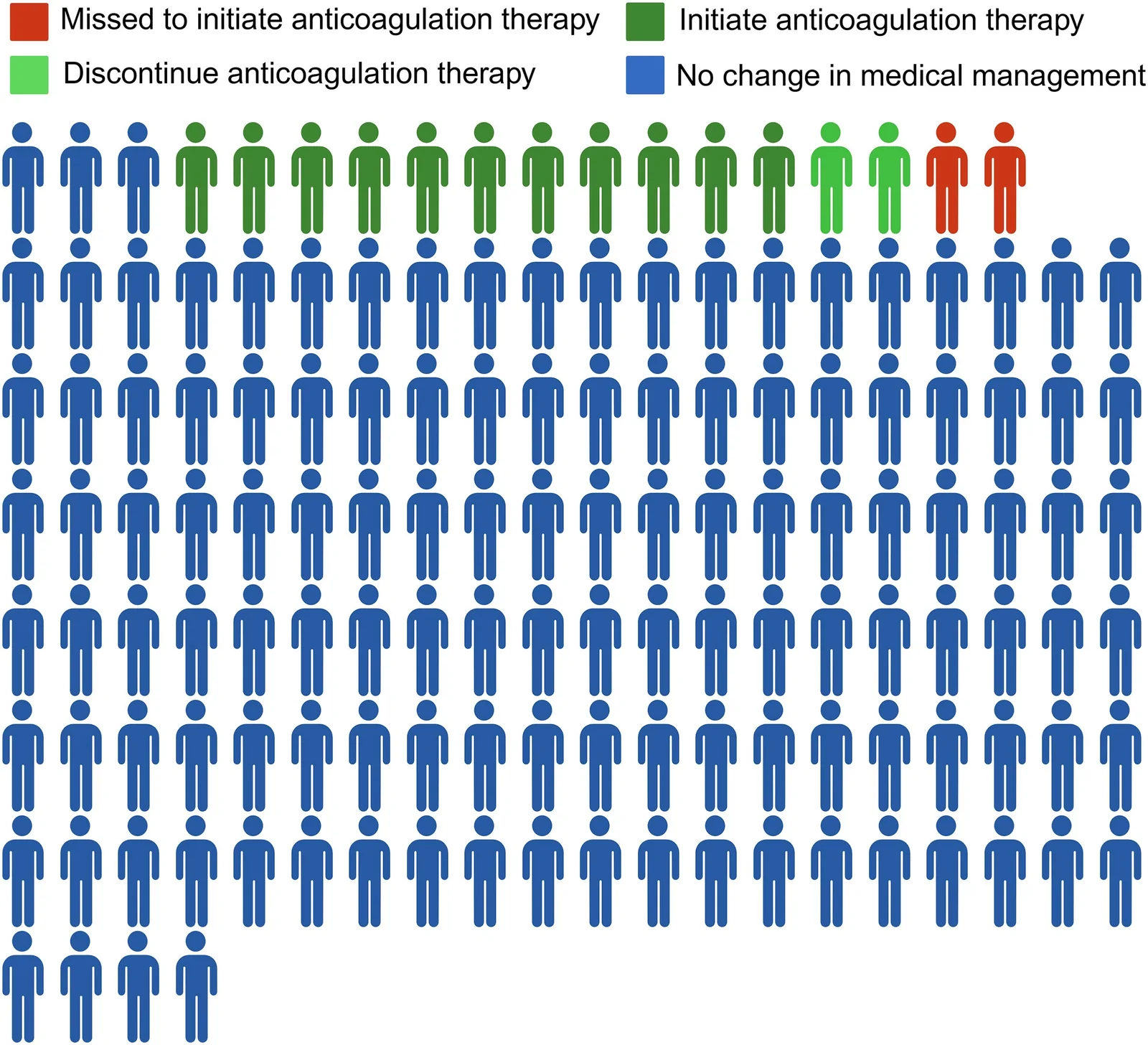
Theoretical treatment implications of large language model (LLM) pipeline-derived CHA_2_DS_2_-VASc recalculation. Theoretical clinical impact of recalculated CHA_2_DS_2_-VASc scores (LLM pipeline using DeepSeek-R1) on anticoagulation management in 142 AF patients with physician-derived CHA_2_DS_2_-VASc scores: 11 patients (dark green) would have been newly and correctly recommended to initiate anticoagulation therapy, while two patients (light green) would have appropriately had therapy discontinued or at least re-evaluated. Two patients who should have received a recommendation for anticoagulation therapy were missed by both the pipeline model and the treating physicians (red).

In an exploratory analysis, we assessed whether the pipeline could identify discrepancies between guideline-recommended and actual treatment. The DeepSeek-R1-powered pipeline model correctly identified 11 patients in the AF cohort who had a clear indication for anticoagulation (i.e. CHA_2_DS_2_-VASc score ≥2) and two patients who might not have had an indication for anticoagulation therapy (i.e. CHA_2_DS_2_-VASc score <2), contrary to the treating physicians’ assessments (*Figure 5*). Furthermore, the model identified four patients with documented diabetes mellitus who lacked a prescription for antidiabetic therapy, highlighting the pipeline’s potential to serve as an automated tool for auditing care quality and optimizing treatment.

### Expert-curated prompting outperforms retrieval-based knowledge injection

Dynamic knowledge retrieval from relevant publications improved the extraction performance for selected score components compared to the standalone LLM approach, as reflected in higher component-level accuracies in the radar plots for variables such as ‘Hypertension’ within the HAS-BLED score (see Supplementary material online, *Figure S2*). However, these improvements did not consistently translate into superior overall score performance. In fact, LLMs with RAG outperformed the standalone LLMs primarily for the EuroSCORE II task, whereas performance for the remaining scores remained comparable or inferior. This apparent advantage for EuroSCORE II is likely attributable to the deterministic post-processing and rule-based score calculation steps integrated into the workflow rather than to consistently improved extraction performance. The inferior performance observed for some score components (see Supplementary material online, *Figure S2*, Supplementary material online, *Figure S3*, Supplementary material online, *Figure S4*) may reflect general limitations of RAG, including prompt dilution^16^ due to increased context length and the retrieval of partially irrelevant or misleading information,^17^ both of which may introduce noise into the extraction process.

Consistent with this interpretation, the RAG-based approach remained substantially inferior to the pipeline approach in both overall agreement and error metrics (*Figure 2*). Given that both approaches shared the same final processing steps, including aggregation and deterministic score calculation, the observed performance gap is most likely attributable to differences in data extraction capabilities.

## Discussion

In summary, we provide the first evidence that an LLM pipeline can calculate cardiovascular risk scores from unstructured, real-world patient data with an accuracy and calibration superior to that of scores documented by treating physicians. Previous studies have highlighted the potential of LLM-based tools for clinical calculations, but their reliance on synthetic vignettes or simplified case reports limited their clinical applicability. Our work directly addresses the calls from those studies to evaluate performance on authentic clinical documents^18,19^ and against the benchmark of real-world human practice,^20,21^ confirming that a thoughtfully architected pipeline can overcome the known limitations of LLMs in this domain.

Recent agent-based approaches such as AgentMD^22^ and RiskAgent^23^ further support the value of combining LLMs with validated calculators and deterministic tool execution. However, these systems differ substantially from our study in both scope and evaluation setting. Whereas AgentMD and RiskAgent emphasize broader agentic tool use and more general risk prediction settings, our work focuses on a transparent, score-specific extraction-plus-computation pipeline for predefined cardiovascular risk scores applied to authentic, heterogeneous clinical documents. We therefore view these approaches as complementary rather than directly comparable.

Importantly, our findings further suggest that successful document-based risk score calculation depends not only on integrating external knowledge sources, tools or calculators, but also on how clinical information is operationalized for extraction. This was reflected in the inferior performance of the RAG-based approach compared with expert-crafted prompts in the pipeline approach. While retrieval from relevant publications provided useful background information, it lacked the structured, task-specific guidance embedded in expert-designed instructions. The expert-designed prompts implicitly capture clinical reasoning heuristics and decision rules that are rarely explicitly formalized in the literature but are critical for interpreting heterogeneous, ambiguous real-world clinical documentation. This is consistent with previous work from our group, in which LLM performance in guideline-concordant treatment decision-making was limited when models were prompted directly with original clinical reports, but improved substantially after clinical information was manually curated.^24^ Together, these findings indicate that fully autonomous retrieval- or agent-based approaches may remain insufficient for robust document-based risk score calculation without human-supervised extraction guidance, particularly for clinically complex multivariable scores.

Another significant finding of our study was the substantial discrepancy between physician-documented scores and the expert-adjudicated ground truth, with treating physicians frequently underestimating patient risk (see Supplementary material online, *Table S6*). While the precise causes are multifactorial, several explanations are plausible. First, clinicians may have an incomplete recall of the specific, nuanced definitions of score components, a known source of miscalculation.^25,26^ Second, in fast-paced clinical environments, time constraints may lead to the reuse of outdated scores from previous encounters, a practice that, while pragmatic, contravenes guideline recommendations for regular risk reassessment.^13^ Similarly, time constraints in routine clinical practice likely explain the observation that, despite explicit guideline recommendations, the HAS-BLED score was calculated in only 20.1% of patients within the AF cohort. While it could be argued that physicians may have had access to additional clinical information not captured in the written records, this is unlikely to account for the observed discrepancy. Such undocumented risk factors would typically lead to higher documented scores. In contrast, we observed that the scores documented by treating physicians were, on average, lower than the ground truth scores (see Supplementary material online, *Table S1*, Supplementary material online, *Table S2*, Supplementary material online, *Table S6*).

The successful performance of our pipeline models suggests that such systems could be integrated into clinical decision support tools to automate and standardize risk assessment. By decoupling language understanding from mathematical computation, our approach provides a blueprint for leveraging the strengths of LLMs while mitigating their weaknesses. This may remove a key historical barrier in clinical predictive modelling: the reliance on simple, manually calculable risk scores.^27^ The ability of LLMs to perform automated, granular data extraction from free-text could unlock the use of more sophisticated and potentially more accurate predictive models that incorporate a far richer set of variables, including narrative-based data that are currently inaccessible to traditional algorithms.

Beyond risk scoring, the pipeline methodology shows promise for broader applications aimed at improving clinical workflow efficiency and patient safety. As our exploratory analysis demonstrated, such a system could be used to audit care and flag potential treatment gaps, such as patients with an indication for anticoagulation or antidiabetic therapy who were not receiving appropriate treatment. This capability could support tasks ranging from identifying clinical trial candidates to compiling data for multidisciplinary heart team or tumour board reviews.

### Limitations

Our study has several limitations. First, its retrospective, single-centre design limits generalizability and requires validation in external cohorts. Second, the pipeline relies on expert-crafted prompts, a manual process that can hinder scalability and carries a risk of partial adaptation to local documentation patterns, potentially reducing generalizability to other institutions and documentation styles. Third, the cross-lingual setup using German input and English prompts may have introduced variability in performance across models, potentially influencing comparative results. Finally, although ground truth was established through a rigorous three-expert consensus, it remains a proxy based on retrospective document review and is subject to epistemic uncertainty due to inherent subjectivity, especially for variables such as NYHA class, representing a limitation for both human and AI assessment. This underlying clinical ambiguity may be a primary driver of the residual errors observed in the pipeline, rather than these errors being attributable solely to model-specific limitations. Given that the analysis relied on documented scores without systematic recalculation under controlled conditions, the comparison between clinician-derived and LLM-based cardiovascular risk score assessments should be interpreted in the context of routine clinical practice rather than optimal physician performance.

## Conclusion

This study provides a validated framework for using an LLM-based pipeline to perform clinical risk scoring from real-world, unstructured data. We show that this approach is not only feasible but can exceed the accuracy of scores documented during routine clinical care. Our findings suggest that approaches such as ours could meaningfully reduce physician workload by automating complex score calculations and improving the consistency and reliability of clinical risk stratification. At the same time, the study highlights that achieving high performance still requires substantial clinical expertise, particularly for the development of expert-curated prompts that operationalize medical reasoning into explicit extraction instructions. Providing models with scientific literature alone appears insufficient to achieve comparable performance.

From a broader perspective, our approach may provide a blueprint for LLM-based clinical decision-support systems and may inform future agentic applications that extract, structure, and operationalize information from routine clinical documentation to support more scalable, data-driven, and evidence-based care.

## Supplementary Material

ztag124_Supplementary_Data

## Data Availability

The (anonymized) data underlying this article will be shared on reasonable request to the corresponding author.

## Code availability

The full analysis pipeline is available as an MCP server application at https://github.com/charite-iaim/mcp-risk-server. All codes, including prompt templates for HAS-BLED, CHA_2_DS_2_-VASc, and EuroSCORE II located in mcp-risk-server/src/prompts, are released under the GNU General Public License v3.0 (GPLv3). The license file and terms are included in the repository; see https://www.gnu.org/licenses/gpl-3.0.html for full details. Users may freely use, modify, and distribute the code and templates, provided derivative works also remain under GPLv3.
