# Supplementary material for: Development of an LLM pipeline exceeding physician-documented cardiovascular risk scores under routine clinical conditions
Source: Eur Heart J Digit Health. 2026 Jul 28;7(7):ztag124. doi: 10.1093/ehjdh/ztag124 (PMC13492338; doi:10.1093/ehjdh/ztag124)
Supplement: Supplementary file 1 [file ztag124_supplementary_data.zip › EHJ-DH-D-25-00451_RevisedManuscript_Supplementary_07.08.26.docx]

Supplementary Material for “Development of an LLM Pipeline Exceeding Physician-documented Cardiovascular Risk Scores under routine clinical conditions”

**AUTHORS**

*Tobias Roeschl, MD^1,2,3,4,5,6^, *Marie Hoffmann, PhD^2,3,5,6^, Axel Unbehaun, MD^2,6^, Henryk Dreger, MD^2,7^, Gerhard Hindricks, MD^1,2^, Volkmar Falk, MD^2,5,6,8^, Ran Balicer^2,9^, Radu Tanacli^2,7^, ^‡^Felix Hohendanner^1,2,7^, ^‡^Alexander Meyer, MD^2,3,4,5,10^

**Corresponding author**: Tobias Roeschl; Charité – Universitätsmedizin Berlin, Institute for Artificial Intelligence in Medicine, Invalidenstraße 120, 10115 Berlin, Germany; [tobias.roeschl@dhzc-charite.de](mailto:tobias.roeschl@dhzc-charite.de)

# Supplementary Material

Prompt templates are provided in EHJ-DH-D-25-00451_Supplementary_prompt_templates.zip.

## Supplementary Figures

Supplementary Figure 1: Calibration of physician-derived scores and standalone LLM-derived scores

Calibration was visualized using kernel density estimation plots, with the ground truth score on the x-axis and the predicted score on the y-axis. The diagonal dashed line indicates perfect calibration. Densities closer to this line reflect better calibration between predicted and true scores.

Supplementary Figure 2: Per-component accuracy for HAS-BLED

Radar plots represent mean accuracies for individual score components. Mean accuracies across all LLMs and components are 0.930 for standalone LLM, 0.863 for LLM with RAG, and 0.993 for the LLM pipeline approach. Score component abbreviations are shown in Supplementary Table 4.

Supplementary Figure 3: Per-component accuracy for CHA_2_DS_2_-VASc

Radar plots represent mean accuracies for individual score components. Mean accuracies across all LLMs and components are 0.917 for standalone LLM, 0.875 for LLM with RAG, and 0.974 for the LLM pipeline approach. Score component abbreviations are shown in Supplementary Table 4.

Supplementary Figure 4: Per-component accuracy for EuroSCORE II

Radar plots represent mean accuracies for individual score components. Mean accuracies across all LLMs and components are 0.919 for standalone LLM, 0.916 for LLM with RAG, and 0.950 for the LLM pipeline approach. Score component abbreviations are shown in Supplementary Table 4.

Supplementary Figure 5: Arithmetic consistency of standalone LLM-derived risk scores

Comparison between final risk scores directly predicted by standalone LLMs and scores recalculated deterministically from the score components reported in the same model outputs. This analysis assesses whether final model outputs were arithmetically consistent with their own component-level predictions. Left panels show the root mean squared error (RMSE) between directly predicted and recalculated scores, while right panels display the exact agreement between both approaches. For HAS-BLED and CHA₂DS₂-VASc, exact agreement required identical scores. For EuroSCORE II, agreement was defined using an absolute tolerance margin of ±0.5 percentage points.

Supplementary Figure 6: Calibration of physician-derived scores and LLM with RAG-derived scores

Calibration was visualized using kernel density estimation plots, with the ground truth score on the x-axis and the predicted score on the y-axis. The diagonal dashed line indicates perfect calibration. Densities closer to this line reflect better calibration between predicted and true scores.

## Supplementary Tables

| **Score component** | **Data availability (%)** | **Summary statistic** |
| --- | --- | --- |
| Age (years) | 100.0 | 69.9 (61.5, 74.5) |
| Documented HAS-BLED score | 20.1 | 1.4 [1.3, 1.5] |
| HAS-BLED score (ground truth) | 100.0 | 1.0 (0.0, 2.0) |
| Uncontrolled hypertension | 100.0 | 13 (7.3%) |
| Renal disease | 100.0 | 0 (0.0%) |
| Liver disease | 100.0 | 0 (0.0%) |
| Stroke history | 100.0 | 17 (9.5%) |
| Bleeding history or predisposition | 100.0 | 25 (14.0%) |
| Labile INR | 100.0 | 0 (0.0%) |
| Elderly (>65 years) | 100.0 | 117 (65.4%) |
| Drugs | 100.0 | 10 (5.6%) |
| Alcohol | 100.0 | 4 (2.2%) |
| Documented CHA_2_DS_2_-VASc score | 79.3 | 2.6 [2.4, 2.8] |
| CHA_2_DS_2_-VASc score (ground truth) | 100.0 | 3.0 (2.0, 4.0) |
| CHF history | 100.0 | 134 (74.9%) |
| Hypertension | 100.0 | 123 (68.7%) |
| Age ≥75 years | 100.0 | 43 (24.0%) |
| Diabetes mellitus | 100.0 | 28 (15.6%) |
| Vascular disease history | 100.0 | 37 (20.7%) |
| Age 65-74 years | 100.0 | 73 (40.8%) |
| Female sex | 100.0 | 67 (37.4%) |
| Systolic blood pressure (mmHg) | 55.9 | 134.5 [131.3, 137.7] |
| LVEF (%) | 77.1 | 55.3 [53.9, 56.7] |
| Hemoglobin (g/dl) | 78.2 | 14.1 [13.9, 14.3] |
| NTproBNP (pg/ml) | 63.1 | 1,264.3 [1044.3, 1484.3] |
| HbA1c (%) | 76.0 | 5.8 [5.7, 5.9] |
| Serum creatinine (mg/dl) | 78.8 | 1.0 [1.0, 1.0] |
| Aspartate transaminase (U/l) | 77.7 | 28.4 [26.7, 30.1] |
| Total bilirubin (mg/dl) | 2.2 | 1.0 [0.9, 1.1] |

Supplementary Table 1: Baseline characteristics of patients with symptomatic atrial fibrillation (n=179)

Patient characteristics were reported as counts and percentages for binary variables, as means with 95% confidence intervals (square brackets) for normally distributed continuous variables, and as medians with interquartile ranges (parentheses) for non-normally distributed variables.

| **Score component** | **Data availability (%)** | **Summary statistic** |
| --- | --- | --- |
| Documented EuroSCORE II | 100.0 | 2.5 (1.6, 3.9) |
| EuroSCORE II (ground truth) | 100.0 | 3.1 (1.8, 4.9) |
| Active endocarditis | 100.0 | 0 (0.0%) |
| CCS angina class 4 | 100.0 | 0 (0.0%) |
| Chronic lung disease | 100.0 | 12 (15.8%) |
| Critical preoperative state | 100.0 | 0 (0.0%) |
| Extracardiac arteriopathy | 100.0 | 17 (22.4%) |
| Female sex | 100.0 | 37 (48.7%) |
| Diabetes on insulin | 100.0 | 8 (10.5%) |
| LV function | 100.0 | good: 59 (77.6%) |
|  |  | moderate: 14 (18.4%) |
|  |  | poor: 3 (3.9%) |
|  |  | very poor: 0 (0.0%) |
| Poor mobility | 100.0 | 7 (9.2%) |
| NYHA class | 100.0 | I: 9 (11.8%) |
|  |  | II: 22 (28.9%) |
|  |  | III: 39 (51.3%) |
|  |  | IV: 6 (7.9%) |
| Pulmonary hypertension | 100.0 | 31-55 mmHg: 38 (50.0%) |
|  |  | >55mmHg: 5 (6.6%) |
|  |  | normal: 33 (43.4%) |
| Recent myocardial infarction | 100.0 | 1 (1.3%) |
| Previous cardiac surgery | 100.0 | 2 (2.6%) |
| Renal impairment | 100.0 | no: 12 (15.8%) |
|  |  | cc 50-85: 46 (60.5%) |
|  |  | on dialysis: 5 (6.6%) |
|  |  | cc <50: 13 (17.1%) |
| eGFR | 90.8 | 64.2 [60.0, 68.4] |
| Surgery on thoracic aorta | 100.0 | 5 (6.6%) |
| Urgency of operation | 100.0 | elective: 74 (97.4%) |
|  |  | urgent: 2 (2.6%) |
|  |  | salvage: 0 (0.0%) |
|  |  | emergency: 0 (0.0%) |
| Weight of operation | 100.0 | isolated CABG 0 (0.0%) |
|  |  | single non-CABG: 61 (80.3%) |
|  |  | two procedures: 15 (19.7%) |
|  |  | three procedures 0 (0.0%) |
| Age (years) | 100.0 | 77.7 [76.1, 79.3] |

Supplementary Table 2: Baseline characteristics of patients with severe aortic stenosis (n=76)

Patient characteristics were reported as counts and percentages for binary variables, as means with 95% confidence intervals (square brackets) for normally distributed continuous variables, and as medians with interquartile ranges (parentheses) for non-normally distributed variables.

| **Score** | **Corpus Category** | **Document** |
| --- | --- | --- |
| **HAS-BLED** | A | Pisters et al.: A novel user-friendly score (HAS-BLED) to assess 1-year risk of major bleeding in patients with atrial fibrillation: the Euro Heart Survey^1^ |
|  | B | Lip et al.: Comparative validation of a novel risk score for predicting bleeding risk in anticoagulated patients with atrial fibrillation^2^ |
|  |  | Lip et al.: Comparative assessment of the HAS-BLED score with other published bleeding risk scoring schemes^3^ |
|  | C | 2020 ESC Guidelines for the Diagnosis and Management of Atrial Fibrillation^4^ |
|  |  | 2016 ESC Guidelines for the Management of Atrial Fibrillation^5^ |
|  | D | Zhu et al.: The HAS-BLED Score for Predicting Major Bleeding Risk in Anticoagulated Patients With Atrial Fibrillation: A Systematic Review and Meta-analysis^6^ |
|  |  | Lip et al.: Bleeding risk assessment and management in atrial fibrillation patients: a position document from the European Heart Rhythm Association, endorsed by the European Society of Cardiology Working Group on Thrombosis^7^ |
|  | E | Ron Pisters. MDCalc. HAS-BLED Score for Major Bleeding Risk. In: MDCalc. Available from: https://www.mdcalc.com/calc/807/has-bled-score-major-bleeding-risk. Accessed March 10, 2026 |
| **CHA_2_DS_2_-VASc** | A | Lip et al.: Refining Clinical Risk Stratification for Predicting Stroke and Thromboembolism in Atrial Fibrillation^8^ |
|  | B | Coppens et al.: CHA₂DS₂-VASc Score study^9^ |
|  |  | van Doorn et al.: Predictive Performance of the CHA₂DS₂-VASc Rule^10^ |
|  | C | 2020 ESC Guidelines for the Diagnosis and Management of Atrial Fibrillation^4^ |
|  |  | 2010 ESC Guidelines for the Management of Atrial Fibrillation^11^ |
|  | D | Jackson et al.: Stroke Risk and Treatment in Patients with Atrial Fibrillation and Low CHA2DS2‐VASc Scores: Findings From the ORBIT‐AF I and II Registries^12^ |
|  | E | Gregory Lip. MDCalc. CHA₂DS₂-VASc Score for Atrial Fibrillation Stroke Risk. In: MDCalc. Available from: https://www.mdcalc.com/calc/801/cha2ds2-vasc-score-atrial-fibrillation-stroke-risk. Accessed March 9, 2026 |
| **EuroSCORE II** | A | Nashef et al.: EuroSCORE II^13^ |
|  | B | Garcia-Valentin et al.: Validation and quality measurements for EuroSCORE and EuroSCORE II in the Spanish cardiac surgical population: a prospective, multicentre study^14^ |
|  |  | Chalmers et al.: Validation of EuroSCORE II in a modern cohort of patients undergoing cardiac surgery^15^ |
|  |  | Heinen et al.: External validation of EuroSCORE I and II in patients with infective endocarditis: results from a nationwide prospective registry^16^ |
|  | C | Neumann et al.: 2018 ESC/EACTS Guidelines on myocardial revascularization^17^ |
|  |  | Vahanian et al.: 2021 ESC/EACTS Guidelines for the management of valvular heart disease^18^ |
|  |  | McDonagh et al.: 2021 ESC Guidelines for the diagnosis and treatment of acute and chronic heart failure^19^ |
|  | D | Argus et al.: Risk Prediction Models for Long-Term Survival after Cardiac Surgery^20^ |
|  | E | EuroSCORE. EuroSCORE II calculator (Available from: <https://www.euroscore.org/index.php?id=17&lang=en>). Cambridge (UK): Royal Papworth Hospital; Accessed March 13, 2026 |

Supplementary Table 3: Composition of the retrieval corpus by evidence category and clinical risk score

Documents included in the retrieval corpus for each clinical risk score, grouped by evidence category: (A) original development studies, (B) validation studies, (C) clinical guidelines, (D) systematic reviews and meta-analyses, and (E) secondary web-based clinical tools.

| **CHA_2_DS_2_-VASc score** | | | |
| --- | --- | --- | --- |
| **Score component** | **Abbreviation** | **Component definition** | **Points awarded** |
| Congestive heart failure | C | Signs and symptoms of congestive heart failure (i.e., dyspnea at rest or on exertion, fatigue, reduced exercise capacity, dizziness, lightheadedness, peripheral edema) and/or a history of decompensated heart failure and/or a history of tachycardia-induced cardiomyopathy and/or a left-ventricular ejection fraction <40% according to the most recent echocardiography exam. | 1 |
| Hypertension | H | Diagnosis of arterial hypertension and/or current antihypertensive pharmacologic treatment | 1 |
| Age ≥75 y | A_2_ | Patient age ≥75 years | 2 |
| Diabetes | D | One or more of the following: Diagnosis of diabetes mellitus, treatment with oral hypoglycemic agents (biguanides, DPP-4 inhibitors, GLP-1 receptor agonists, thiazolidinediones) and/or insulin, HbA1c ≥6.5% | 1 |
| Stroke | S_2_ | Prior stroke and/or prior transient ischemic attack and/or a history of arterial thromboembolism | 2 |
| Vascular | V | One or more of the following: Prior myocardial infarction, significant coronary artery disease (defined as a greater than 50% angiographic narrowing of at least one coronary artery and/or a history of percutaneous coronary intervention), peripheral artery disease, complex aortic plaque | 1 |
| Age 65-74 y | A | Patient age between 65 and 74 years | 1 |
| Sex category | Sc | Female sex | 1 |
| **HAS-BLED score** | | | |
| **Score component** | **Abbreviation** | **Component definition** | **Points awarded** |
| Hypertension | H | Systolic blood pressure >160 mmHg and/or a diagnosis of uncontrolled hypertension | 1 |
| Abnormal renal function | A1 | One or more of the following: Dialysis, kidney transplant, serum creatinine >200 μmol/L (>2.26 mg/dL) | 1 |
| Abnormal hepatic function | A2 | One or more of the following: Liver cirrhosis, total bilirubin >2 x upper limit of normal, aspartate aminotransferase/alanine aminotransferase/alkaline phosphatase >3 × upper limit normal | 1 |
| Stroke | S | Previous ischemic and/or hemorrhagic stroke | 1 |
| Bleeding | B | Previous major hemorrhage (defined as intracranial bleeding and/or bleeding requiring hospitalization and/or blood transfusion and/or immediate therapy such as endoscopy) and/or anemia (defined as hemoglobin level <13 g/dL if male, and <12 g/dL if female) | 1 |
| Labile INR | L | Time in therapeutic range <60% in patients receiving vitamin K antagonists | 1 |
| Elderly | E | Patient age >65 years | 1 |
| Drugs | D1 | Concomitant use of antiplatelet drugs and/or NSAIDs | 1 |
| Alcohol | D2 | Excessive alcohol consumption defined as ≥8 alcoholic drinks/week and/or a diagnosis of alcoholism | 1 |
| **EuroSCORE II** | | | |
| **Score component** | **Abbreviation** | **Component definition** | **Regression coefficient** |
| NYHA class | NYHA | • I: no symptoms on moderate exertion  • II: symptoms on moderate exertion  • III: symptoms on light exertion  • IV: symptoms at rest | 0  0.1070545  0.2958  0.55979 |
| CCS4 | CCS4 | CCS class 4 angina (inability to perform any activity without angina or angina at rest) | 0.2226147 |
| IDDM | IDDM | Insulin-dependent diabetes mellitus | 0.3542749 |
| Age | Age | Patient age in years  • age ≤60  • age >60 | 0.0285181  (age - 59)* 0.0285181 |
| Female sex | Female | Patient is female | 0.2196434 |
| Extracardiac arteriopathy | ECA | One or more of the following: Claudication, carotid occlusion or >50% stenosis, prior amputation for arterial disease, previous or planned intervention on the abdominal aorta, limb arteries or carotids. | 0.5360268 |
| Chronic pulmonary disease | CPD | The patient is on bronchodilators or steroids for lung disease | 0.1886564 |
| Poor mobility | N/M | Severe impairment of mobility secondary to musculoskeletal or neurological dysfunction | 0.2407181 |
| Previous cardiac surgery | Redo | One or more previous major cardiac operations requiring opening of the pericardium. | 1.118599 |
| Renal dysfunction | Renal | • CC >85 ml/min  • CC 50–85 ml/min  • on dialysis (regardless of serum creatinine)  • CC <50 ml/min | 0  0.303553  0.6421508  0.8592256 |
| Active endocarditis | AE | Patients still on antibiotic treatment for endocarditis at the time of surgery. | 0.6194522 |
| Critical preoperative state | Critical | Any one or more of the following occurring preoperatively in the same hospital admission as the operation: ventricular tachycardia or fibrillation or aborted sudden death, cardiac massage, ventilation before arrival in the anaesthetic room, inotropes, intra-aortic balloon counterpulsation or ventricular-assist device before arrival in the anesthetic room, acute renal failure (anuria or oliguria) | 1.086517 |
| LV function | LV | • good (LVEF >50%)  • moderate (LVEF 31–50%)  • poor (LVEF 21–30%)  • very poor (LVEF ≤20%) | 0  0.3150652  0.8084096  0.934691 |
| Recent myocardial infarction | RMI | Myocardial infarction within the last 90 days | 0.1528943 |
| PA systolic pressure | PASP | • <31 mmHg  • 31-55 mmHg  • >55 mmHg | 0  0.1788899  0.3491475 |
| Urgency | Urgency | • elective: routine admission for operation  • urgent: patients not electively admitted for operation but who require surgery on the current admission for medical reasons and cannot be discharged without a definitive procedure  • emergency: operation before the beginning of the next working day after decision to operate  • salvage: patients requiring cardiopulmonary resuscitation (external cardiac massage) en route to the operating theatre or before induction of anesthesia. This does not include cardiopulmonary resuscitation after induction of anesthesia. | 0  0.3174673  0.7039121  1.362947 |
| Weight of procedure | Weight | • isolated CABG  • isolated non-CABG major procedure (e.g. single valve procedure, replacement of ascending aorta, correction of septal defect, etc.)  • two major procedures (e.g. CABG + AVR), or CABG + mitral valve repair (MVR), or AVR + replacement of ascending aorta, or CABG + maze procedure, or AVR + MVR, etc.)  • three major procedures or more (e.g. AVR + MVR + CABG, or MVR + CABG + tricuspid annuloplasty, etc.), or aortic root replacement when it includes AVR or repair + coronary reimplantation + root and ascending replacement). | 0  0.0062118  0.5521478  0.9724533 |
| Thoracic aorta | ThoAo | Disorder of the thoracic aorta requiring surgery | 0.6527205 |
| Constant | - | - | −5.324537 |

Supplementary Table 4: Score component definitions

Score components, their definitions and the respective points/logistic regression coefficients used in our study are shown for the CHA_2_DS_2_-VASc^8^, HAS-BLED^1^ and EuroSCORE II^13^ next to the abbreviations used in Figure 3, Supplementary Figure 2, Supplementary Figure 3, and Supplementary Figure 4.

| **Score** | **Experiment** | **Model** | **α [CI]** | **RMSE [CI]** |
| --- | --- | --- | --- | --- |
| HAS-BLED | Physician | - | -0.06 [-0.15, 0.00] | 0.87 [0.69, 1.04] |
|  | Standalone LLM | GPT-OSS 120B | 0.12 [-0.08, 0.32] | 1.01 [0.90, 1.13] |
|  |  | Kimi K2.5 | 0.24 [0.02, 0.45] | 0.81 [0.70, 0.92] |
|  |  | Qwen3-235B-A22B | -0.03 [-0.19, 0.14] | 1.30 [1.15, 1.46] |
|  |  | DeepSeek-R1 | 0.51 [0.22, 0.74] | 0.61 [0.50, 0.72] |
|  |  | Gemini 3.1 Pro | 0.57 [0.27, 0.80] | 0.49 [0.40, 0.57] |
|  |  | GPT-5.2 | 0.24 [0.01, 0.46] | 0.79 [0.70, 0.88] |
|  | LLM RAG | GPT-OSS 120B | 0.08 [-0.11, 0.27] | 1.04 [0.92, 1.15] |
|  |  | Kimi K2.5 | -0.05 [-0.21, 0.13] | 1.35 [1.23, 1.47] |
|  |  | Qwen3-235B-A22B | -0.25 [-0.39, -0.10] | 1.78 [1.62, 1.92] |
|  |  | DeepSeek-R1 | -0.31 [-0.46, -0.16] | 1.77 [1.64, 1.90] |
|  |  | Gemini 3.1 Pro | 0.40 [0.12, 0.64] | 0.58 [0.51, 0.66] |
|  |  | GPT-5.2 | -0.12 [-0.28, 0.04] | 1.40 [1.29, 1.50] |
|  | LLM Pipeline | GPT-OSS 120B | 0.76 [0.47, 0.95] | 0.20 [0.11, 0.26] |
|  |  | Kimi K2.5 | 0.72 [0.40, 0.92] | 0.25 [0.17, 0.32] |
|  |  | Qwen3-235B-A22B | 0.93 [0.71, 1.00] | 0.20 [0.11, 0.26] |
|  |  | DeepSeek-R1 | 0.81 [0.53, 1.00] | 0.30 [0.22, 0.37] |
|  |  | Gemini 3.1 Pro | 0.87 [0.60, 1.00] | 0.17 [0.07, 0.24] |
|  |  | GPT-5.2 | 0.72 [0.40, 0.92] | 0.25 [0.17, 0.32] |
| CHA_2_DS_2_-VASc | Physician | - | 0.53 [0.29, 0.72] | 1.08 [0.96, 1.20] |
|  | Standalone LLM | GPT-OSS 120B | 0.76 [0.56, 0.92] | 0.81 [0.73, 0.88] |
|  |  | Kimi K2.5 | 0.70 [0.50, 0.86] | 0.86 [0.77, 0.96] |
|  |  | Qwen3-235B-A22B | 0.68 [0.45, 0.85] | 0.89 [0.80, 0.99] |
|  |  | DeepSeek-R1 | 0.74 [0.55, 0.89] | 0.78 [0.71, 0.86] |
|  |  | Gemini 3.1 Pro | 0.70 [0.48, 0.86] | 0.82 [0.72, 0.91] |
|  |  | GPT-5.2 | 0.69 [0.48, 0.85] | 0.82 [0.75, 0.90] |
|  | LLM RAG | GPT-OSS 120B | 0.58 [0.38, 0.75] | 1.07 [0.94, 1.21] |
|  |  | Kimi K2.5 | 0.80 [0.61, 0.94] | 0.93 [0.82, 1.04] |
|  |  | Qwen3-235B-A22B | 0.54 [0.28, 0.74] | 1.04 [0.92, 1.15] |
|  |  | DeepSeek-R1 | 0.60 [0.39, 0.78] | 1.05 [0.94, 1.16] |
|  |  | Gemini 3.1 Pro | 0.72 [0.51, 0.89] | 0.93 [0.83, 1.03] |
|  |  | GPT-5.2 | 0.75 [0.56, 0.90] | 0.99 [0.88, 1.09] |
|  | LLM Pipeline | GPT-OSS 120B | 0.94 [0.83, 1.00] | 0.39 [0.31, 0.47] |
|  |  | Kimi K2.5 | 0.91 [0.78, 1.00] | 0.45 [0.36, 0.54] |
|  |  | Qwen3-235B-A22B | 0.88 [0.74, 0.97] | 0.53 [0.40, 0.66] |
|  |  | DeepSeek-R1 | 0.89 [0.76, 0.97] | 0.48 [0.38, 0.57] |
|  |  | Gemini 3.1 Pro | 0.85 [0.70, 0.96] | 0.65 [0.40, 0.89] |
|  |  | GPT-5.2 | 0.91 [0.78, 1.00] | 0.45 [0.36, 0.55] |
| EuroSCORE II | Physician | - | 0.45 [0.18, 0.69] | 2.05 [1.50, 2.56] |
|  | Standalone LLM | GPT-OSS 120B | -0.43 [-0.61, -0.25] | 21.12 [14.11, 28.02] |
|  |  | Kimi K2.5 | -0.23 [-0.43, -0.03] | 21.90 [15.23, 28.04] |
|  |  | Qwen3-235B-A22B | -0.09 [-0.30, 0.12] | 28.96 [19.07, 37.55] |
|  |  | DeepSeek-R1 | 0.09 [-0.13, 0.30] | 12.14 [9.49, 14.65] |
|  |  | Gemini 3.1 Pro | 0.57 [0.36, 0.74] | 1.70 [1.35, 2.06] |
|  |  | GPT-5.2 | -0.01 [-0.26, 0.22] | 8.81 [6.09, 11.33] |
|  | LLM RAG | GPT-OSS 120B | 0.62 [0.42, 0.78] | 1.55 [1.19, 1.89] |
|  |  | Kimi K2.5 | 0.43 [0.21, 0.62] | 3.90 [2.47, 5.27] |
|  |  | Qwen3-235B-A22B | 0.48 [0.27, 0.67] | 6.09 [4.11, 7.89] |
|  |  | DeepSeek-R1 | 0.60 [0.40, 0.76] | 2.44 [1.77, 3.19] |
|  |  | Gemini 3.1 Pro | 0.50 [0.27, 0.71] | 1.83 [1.38, 2.26] |
|  |  | GPT-5.2 | 0.59 [0.39, 0.75] | 3.59 [1.76, 5.37] |
|  | LLM Pipeline | GPT-OSS 120B | 0.62 [0.42, 0.79] | 1.90 [1.32, 2.47] |
|  |  | Kimi K2.5 | 0.64 [0.44, 0.79] | 2.27 [1.35, 3.20] |
|  |  | Qwen3-235B-A22B | 0.72 [0.55, 0.87] | 2.83 [1.41, 4.35] |
|  |  | DeepSeek-R1 | 0.58 [0.36, 0.75] | 1.38 [1.05, 1.69] |
|  |  | Gemini 3.1 Pro | 0.68 [0.50, 0.83] | 1.74 [0.85, 2.68] |
|  |  | GPT-5.2 | 0.61 [0.40, 0.78] | 1.61 [1.20, 2.01] |

Supplementary Table 5: Performance metrics

Krippendorff's alpha and root mean squared error (RMSE) for physician-documented scores, standalone LLMs, LLMs with RAG, and LLM pipelines with 95% confidence intervals.

| **Score** | **N** | **Mean differences** |
| --- | --- | --- |
| HAS-BLED score | 36 | -0.14 [-0.43, 0.15] |
| CHA_2_DS_2_-VASc score | 142 | 0.64 [0.5, 0.78] |
| EuroSCORE II | 76 | 0.61 [0.16, 1.06] |

Supplementary Table 6: Mean difference between ground truth and physician-derived scores with 95% confidence intervals

Mean differences were calculated by subtracting physician-derived scores from ground-truth scores for each case and then averaging the resulting differences.

N denotes the number of samples with non-missing scores (i.e., available data) used in the analysis.

| **Variable** | **Agreement** | **Raw Agreement** |
| --- | --- | --- |
| **HAS-BLED score** | 0.85 | 0.99 |
| Hypertension | 0.88 | 0.98 |
| Abnormal renal function | - | 1.0 |
| Abnormal liver function | - | 0.99 |
| Stroke | 0.64 | 0.96 |
| Bleeding | 0.85 | 0.99 |
| Labile INR | - | 1.0 |
| Elderly (age >65 years) | 0.95 | 0.98 |
| Drugs | 0.88 | 0.99 |
| Alcohol | - | 0.97 |
| **CHA_2_DS_2_-VASc score** | 0.97 | 0.99 |
| Congestive heart failure | 0.77 | 0.91 |
| Hypertension | 0.91 | 0.96 |
| Age ≥75 years | 1.0 | 1.0 |
| Diabetes mellitus | 0.95 | 0.99 |
| Stroke | 0.96 | 0.99 |
| Vascular disease | 0.93 | 0.98 |
| Age (65-74 years) | 0.99 | 0.99 |
| Sex category (female) | 1.0 | 1.0 |
| **EuroSCORE II** | 0.94 | 0.97 |
| Active endocarditis | - | 1.0 |
| CCS IV | - | 0.96 |
| Chronic pulmonary disease | 0.72 | 0.92 |
| Critical preoperative state | - | 1.0 |
| Extracardiac arteriopathy | 0.72 | 0.91 |
| Female sex | 0.97 | 0.99 |
| Insulin-dependent diabetes mellitus | 1.0 | 1.0 |
| LV function | 0.90 | 0.95 |
| Poor mobility | - | 0.99 |
| NYHA class | 0.42 | 0.57 |
| PA systolic pressure | 0.81 | 0.88 |
| Recent myocardial infarction | - | 0.97 |
| Previous cardiac surgery | 0.66 | 0.97 |
| Renal dysfunction | 0.68 | 0.96 |
| Thoracic aorta | 0.55 | 0.96 |
| Urgency | 0.25 | 0.88 |
| Weight of procedure | 0.63 | 0.93 |

Supplementary Table 7: Interrater Agreement Metrics Among Clinical Raters

Krippendorff’s alpha is reported for each variable where sufficient variability across categories allowed its calculation. For variables with sparse data—defined as any category represented by a single observation—only raw agreement is presented due to the instability or inapplicability of standard reliability coefficients. Abbreviations as in Supplementary Table 4.

## Supplementary References

1.	Pisters R, Lane DA, Nieuwlaat R, de Vos CB, Crijns HJGM, Lip GYH. A Novel User-Friendly Score (HAS-BLED) To Assess 1-Year Risk of Major Bleeding in Patients With Atrial Fibrillation: The Euro Heart Survey. *Chest*. 2010/11/01/ 2010;138(5):1093–1100. doi:<https://doi.org/10.1378/chest.10-0134>

2.	Lip GY, Frison L, Halperin JL, Lane DA. Comparative validation of a novel risk score for predicting bleeding risk in anticoagulated patients with atrial fibrillation: the HAS-BLED (Hypertension, Abnormal Renal/Liver Function, Stroke, Bleeding History or Predisposition, Labile INR, Elderly, Drugs/Alcohol Concomitantly) score. *J Am Coll Cardiol*. Jan 11 2011;57(2):173–80. doi:10.1016/j.jacc.2010.09.024

3.	Lip GY, Lin HJ, Hsu HC, et al. Comparative assessment of the HAS-BLED score with other published bleeding risk scoring schemes, for intracranial haemorrhage risk in a non-atrial fibrillation population: the Chin-Shan Community Cohort Study. *Int J Cardiol*. Oct 3 2013;168(3):1832–6. doi:10.1016/j.ijcard.2012.12.076

4.	Hindricks G, Potpara T, Dagres N, et al. 2020 ESC Guidelines for the diagnosis and management of atrial fibrillation developed in collaboration with the European Association for Cardio-Thoracic Surgery (EACTS): The Task Force for the diagnosis and management of atrial fibrillation of the European Society of Cardiology (ESC) Developed with the special contribution of the European Heart Rhythm Association (EHRA) of the ESC. *European Heart Journal*. 2020;42(5):373–498. doi:10.1093/eurheartj/ehaa612

5.	Kirchhof P, Benussi S, Kotecha D, et al. 2016 ESC Guidelines for the management of atrial fibrillation developed in collaboration with EACTS. *Eur Heart J*. Oct 7 2016;37(38):2893–2962. doi:10.1093/eurheartj/ehw210

6.	Zhu W, He W, Guo L, Wang X, Hong K. The HAS-BLED Score for Predicting Major Bleeding Risk in Anticoagulated Patients With Atrial Fibrillation: A Systematic Review and Meta-analysis. *Clin Cardiol*. Sep 2015;38(9):555–61. doi:10.1002/clc.22435

7.	Lip GY, Andreotti F, Fauchier L, et al. Bleeding risk assessment and management in atrial fibrillation patients: a position document from the European Heart Rhythm Association, endorsed by the European Society of Cardiology Working Group on Thrombosis. *Europace*. May 2011;13(5):723–46. doi:10.1093/europace/eur126

8.	Lip GY, Nieuwlaat R, Pisters R, Lane DA, Crijns HJ. Refining clinical risk stratification for predicting stroke and thromboembolism in atrial fibrillation using a novel risk factor-based approach: the euro heart survey on atrial fibrillation. *Chest*. Feb 2010;137(2):263–72. doi:10.1378/chest.09-1584

9.	Coppens M, Eikelboom JW, Hart RG, et al. The CHA2DS2-VASc score identifies those patients with atrial fibrillation and a CHADS2 score of 1 who are unlikely to benefit from oral anticoagulant therapy. *European Heart Journal*. 2012;34(3):170–176. doi:10.1093/eurheartj/ehs314

10.	van Doorn S, Debray TPA, Kaasenbrood F, et al. Predictive performance of the CHA2DS2‐VASc rule in atrial fibrillation: a systematic review and meta‐analysis. *Journal of Thrombosis and Haemostasis*. 2017/06/01/ 2017;15(6):1065–1077. doi:<https://doi.org/10.1111/jth.13690>

11.	Camm AJ, Kirchhof P, Lip GY, et al. Guidelines for the management of atrial fibrillation: the Task Force for the Management of Atrial Fibrillation of the European Society of Cardiology (ESC). *Eur Heart J*. Oct 2010;31(19):2369–429. doi:10.1093/eurheartj/ehq278

12.	Jackson LR, Kim S, Fonarow GC, et al. Stroke Risk and Treatment in Patients with Atrial Fibrillation and Low CHA₂DS₂-VASc Scores: Findings From the ORBIT-AF I and II Registries. J Am Heart Assoc. 2018;7(16):e008764. doi:10.1161/JAHA.118.008764

13.	Nashef SA, Roques F, Sharples LD, et al. EuroSCORE II. *Eur J Cardiothorac Surg*. Apr 2012;41(4):734–44; discussion 744–5. doi:10.1093/ejcts/ezs043

14.	Garcia-Valentin A, Mestres CA, Bernabeu E, et al. Validation and quality measurements for EuroSCORE and EuroSCORE II in the Spanish cardiac surgical population: a prospective, multicentre study †. *European Journal of Cardio-Thoracic Surgery*. 2015;49(2):399–405. doi:10.1093/ejcts/ezv090

15.	Chalmers J, Pullan M, Fabri B, et al. Validation of EuroSCORE II in a modern cohort of patients undergoing cardiac surgery. *European Journal of Cardio-Thoracic Surgery*. 2012;43(4):688–694. doi:10.1093/ejcts/ezs406

16.	Heinen FJ, Peijster AJL, Fu EL, et al. External validation of EuroSCORE I and II in patients with infective endocarditis: results from a nationwide prospective registry. *European Journal of Cardio-Thoracic Surgery*. 2024;66(6)doi:10.1093/ejcts/ezae418

17.	Neumann F-J, Sousa-Uva M, Ahlsson A, et al. 2018 ESC/EACTS Guidelines on myocardial revascularization. *European Heart Journal*. 2018;40(2):87–165. doi:10.1093/eurheartj/ehy394

18.	Vahanian A, Beyersdorf F, Praz F, et al. 2021 ESC/EACTS Guidelines for the management of valvular heart disease. *Eur Heart J*. Feb 12 2022;43(7):561–632. doi:10.1093/eurheartj/ehab395

19.	McDonagh TA, Metra M, Adamo M, et al. 2021 ESC Guidelines for the diagnosis and treatment of acute and chronic heart failure: Developed by the Task Force for the diagnosis and treatment of acute and chronic heart failure of the European Society of Cardiology (ESC) With the special contribution of the Heart Failure Association (HFA) of the ESC. *European Heart Journal*. 2021;42(36):3599–3726. doi:10.1093/eurheartj/ehab368

20.	Argus L, Taylor M, Ouzounian M, Venkateswaran R, Grant SW. Risk Prediction Models for Long-Term Survival after Cardiac Surgery: A Systematic Review. *Thorac Cardiovasc Surg*. Jan 2024;72(1):29–39. doi:10.1055/s-0043-1760747
